# Cracking the LUTS Code: A Pre-Urodynamic Tool for DU vs. BOO Diagnosis in Female Patients with Non-Neurogenic LUTS

**DOI:** 10.3390/jcm14113674

**Published:** 2025-05-23

**Authors:** Karolina Garbas, Łukasz Zapała, Aleksander Ślusarczyk, Tomasz Piecha, Piotr Radziszewski

**Affiliations:** 1 Department of General, Oncological and Functional Urology, Medical University of Warsaw, 02-005 Warsaw, Poland; 2 Urodynamic Lab of Private Hospital “Prostalith”, 25-613 Kielce, Poland

**Keywords:** detrusor underactivity, female bladder outlet obstruction, LUTS, non-invasive diagnostic methods, uroflowmetry

## Abstract

**Background**: Detrusor underactivity (DU) and bladder outlet obstruction (BOO) are common causes of voiding dysfunction in women with lower urinary tract symptoms (LUTS). However, differentiating between them remains challenging due to overlapping clinical presentations and a reliance on invasive urodynamic studies (UDS). This study aimed to develop a non-invasive, office-based clinical prediction model to distinguish DU from BOO in women with non-neurogenic LUTS. **Methods**: We conducted a retrospective analysis of 88 women who underwent pressure-flow studies at two outpatient clinics between 2012 and 2022. DU was defined using a projected isovolumetric pressure 1 (PIP1) < 30 cm H_2_O, and BOO was defined by a Female-Specific Bladder Outlet Obstruction Index (BOOIf) > 18. Clinical symptoms, uroflowmetry (UFL) parameters, and pelvic organ prolapse staging (POP-Q) were evaluated. A multivariate logistic regression model was constructed using a stepwise selection procedure. **Results**: Of the 88 patients, 38 (43.2%) were diagnosed with DU and 50 (56.8%) with BOO. Four predictors were retained in the final model: hesitancy (OR = 2.06, *p* = 0.18), incomplete emptying (OR = 3.52, *p* = 0.02), POP-Q < 3 (OR = 0.15, *p* = 0.02), and longer time to Qmax on UFL (OR = 1.05, *p* = 0.004). The model achieved a Harrell’s Concordance Index (C-index) of 0.779. Using a probability cutoff of 0.3, the model demonstrated a sensitivity of 86.8%, specificity of 46.0%, positive predictive value of 55.0%, and negative predictive value of 82.1%. **Conclusions**: We present a novel non-invasive prediction model incorporating clinical symptoms, UFL metrics, and pelvic exam findings that may aid in differentiating DU from BOO in women with LUTS.

## 1. Introduction

Detrusor underactivity (DU) is increasingly recognized as a significant cause of voiding dysfunction (VD) in both men and women. However, data on female DU remain limited and, in some cases, contradictory. The reported prevalence rates of DU vary depending on the study population. Among women with lower urinary tract symptoms (LUTS) without cystocele, DU has been diagnosed in approximately 10% [1] to 17% [2] of cases. Furthermore, evidence suggests that the prevalence of DU increases with age [2]. Accurately determining its true prevalence is challenging due to the long-standing absence of standardized diagnostic criteria. In women attending pelvic floor clinics, DU prevalence varied substantially depending on the applied definition: 33.7% according to Schäfer’s detrusor factor, 37.0% using Abrams’ Bladder Contractility Index (BCI), and 4.1% based on Jeong’s criteria [3].

The International Continence Society (ICS) defines DU as a *“detrusor contraction of reduced strength and/or duration, resulting in prolonged bladder emptying and/or a failure to achieve complete bladder emptying within a normal time span”* [4]. Bladder outlet obstruction, which can present with similar clinical symptoms, is defined as *“the obstruction during voiding, (…) a reduced urinary flow rate and/or presence of a raised post-void residual and an increased detrusor pressure. (…) A urethral stricture or obstruction due to higher degrees of uterovaginal prolapse or obstructed voiding after stress incontinence procedures are among possible causes”* [4].

Distinguishing DU from BOO remains diagnostically challenging, as the symptom overlap is considerable. Currently, urodynamic studies (UDS) are considered the gold standard for evaluating female VD, including DU and BOO [5]. However, until recently, the urodynamic criteria for diagnosing DU in women lacked precision and consistency.

Recent recommendations from the ICS and the Society of Urodynamics, Female Pelvic Medicine & Urogenital Reconstruction (SUFU) strongly advise against using the BCI and its updated version—the ICS-Detrusor Contractility Index (ICS-DCI)—as a standard metric for female VD due to insufficient validation, particularly for diagnosing DU in individual women. While an ICS-DCI value greater than 100 is generally accepted as indicative of adequate detrusor voiding contraction (DVC), the clinical significance of ICS-DCI values below 100 remains unclear [6]. A study assessing isovolumetric contraction pressures found that the Detrusor/Bladder Contractility Index (D[B]CI) significantly overestimates isovolumetric (“stop-test”) detrusor pressures in women. In contrast, projected isovolumetric pressure 1 (PIP1 = PdetQmax + Qmax) has been shown to provide a more reliable estimate [7]. A PIP1 value below 30 cm H_2_O has been proposed as the lower limit for normal DVC [6]. For the diagnosis of BOO in females, a Female-Specific Bladder Outlet Obstruction Index (BOOIf) has been introduced, calculated as PdetQmax—2.2Qmax. A BOOIf value exceeding 18 has been identified as a strong predictor of BOO [8] and has been incorporated into the provisional ICS pressure-flow study (ICS-PFS) nomogram for women [6].

Despite these advancements in invasive diagnostics, no widely accepted non-invasive predictive model currently exists to differentiate DU from BOO in women. Therefore, the objective of this pilot study was to develop a first-stage, non-invasive, office-based model to support the diagnosis of DU in women with non-neurogenic LUTS and to differentiate it from BOO. This approach is intended to assist clinicians in making more efficient, evidence-informed decisions in the diagnostic workup of voiding dysfunction.

## 2. Materials and Methods

### 2.1. Patient Selection and Data Collection

We conducted a retrospective analysis of clinical records from 3161 patients who underwent pressure-flow assessments between 2012 and 2022 at two outpatient facilities. Individuals were referred for urodynamic testing due to new or persistent LUTS, unsuccessful conservative management, preoperative evaluation for urological or gynecological procedures, failure of previous invasive interventions, or preparation for kidney transplantation. Prior to undergoing pressure-flow studies (PFS), all patients had presented negative urine cultures confirming the absence of urinary tract infections. Each patient underwent a standard clinical evaluation, which included a thorough medical history covering comorbidities and current medications, completion of the Core Lower Urinary Tract Symptoms (CLSS) questionnaire [9], and a basic neurological examination [10], as previously described [11]. A urogenital examination was performed on every woman, and pelvic organ prolapse was staged according to the Pelvic Organ Prolapse Quantification (POP-Q) system [12]. Following these assessments, both a free-flow test and a PFS were performed.

**Figure 1:**
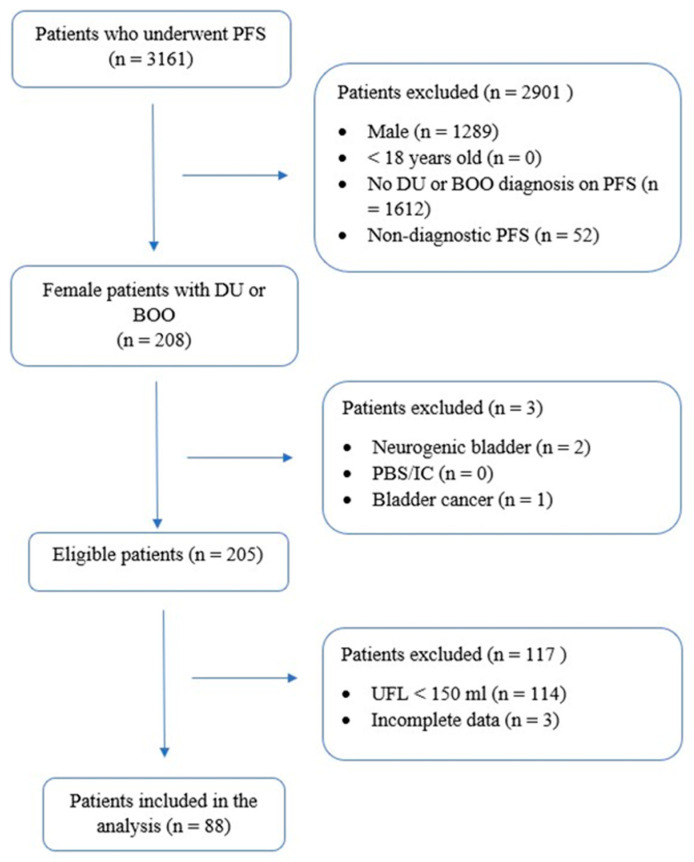
Flow chart of patients included in the final analysis. PFS—pressure-flow study, DU—detrusor underactivity, BOO—bladder outlet obstruction, PBS/IC—painful bladder syndrome/interstitial cystitis, UFL—uroflowmetry.

The inclusion criteria for the study required participants to be adult females experiencing LUTS and diagnosed with either DU or BOO based on pressure-flow findings. Additionally, participants had to provide informed consent, undergo UFL with a voided volume exceeding 150 mL, and then subsequently undergo PFS. Patients were excluded from the study if they had a history of neurogenic bladder, painful bladder syndrome/interstitial cystitis (PBS/IC), bladder cancer, or if their PFS results were inconclusive (e.g., patient-interrupted tests, low voided volume, or displacement of bladder or rectal catheter during examination). Incomplete medical records also led to exclusion. Ultimately, 88 women met all criteria and were included in the final analysis (Figure 1). The study received approval from the Bioethics Committee of the Medical University of Warsaw (approval no. AKBE/335/2023).

The evaluated UFL parameters included maximum flow rate (Qmax), average flow rate (Qav), voided volume, time to reach Qmax, post-void residual urine (PVR), and flow-curve shape. Flow curves were categorized into predefined patterns, including bell-shaped, fluctuating, intermittent, fluctuating-intermittent, plateau, or atypical (not conforming to the aforementioned categories). Post-void residual urine was quantified via abdominal ultrasound following UFL, and the post-void residual ratio (PVR-R) was determined as the proportion of the PVR to total bladder volume (sum of voided volume and PVR). The PFS was conducted after UFL by urodynamic-trained experts in adherence to the ICS Good Urodynamics Practices protocol [13]. DU was identified based on a Projected Isovolumetric Pressure 1 (PIP1) calculation, using the formula: PIP1 = PdetQmax + Qmax [6]. BOO was diagnosed if the Female BOO Index (BOOIf), calculated using the equation BOOI = PdetQmax − 2.2Qmax, exceeded 18 [6]. The assessments were performed using Medtronic equipment (Duet Logic G/2, model: 9032A0173, Medtronic, Lublin, Poland). All recorded data were stored within the Medtronic software version 1.2 system and analyzed in accordance with ICS guidelines [4]. Each PFS underwent rigorous manual verification to identify and eliminate potential artifacts, ensuring data integrity. All validated findings were manually entered into the study database.

### 2.2. Statistical Analysis

Baseline demographic and clinical characteristics were summarized using medians with interquartile ranges for continuous variables, while categorical variables were reported as numbers with corresponding percentages. Comparisons of continuous variables were conducted using the Mann–Whitney U test, whereas categorical data were analyzed using Fisher’s exact test. To assess associations between variables, logistic regression was employed for both univariate and multivariate analyses. A multivariable analysis was carried out using a stepwise selection approach, where variables identified as relevant in univariate analyses were systematically introduced into the model. The stepwise selection process involved adding variables to the model based on an inclusion threshold of 0.2. Once incorporated, each variable was reassessed at every stage, and those with a *p*-value exceeding 0.2 were removed, ensuring that only predictors contributing to model optimization remained. This process continued iteratively until no further refinement was possible, and only statistically significant predictors were retained in the final model. Odds ratios with 95% confidence intervals were estimated through logistic regression. To evaluate the model’s performance, sensitivity, specificity, positive predictive value (PPV), and negative predictive value (NPV) were calculated. The model’s diagnostic accuracy was assessed using a probability cutoff of 0.3. Statistical significance was determined using two-sided *p*-values, with a threshold of <0.05. All statistical analyses were conducted using SAS software (version 9.4, SAS Institute Inc., Cary, NC, USA). Given the limited sample size and preliminary scope, this study should be considered a pilot investigation aimed at generating hypotheses and informing the design of future validation studies.

## 3. Results

### 3.1. Baseline Characteristics

Eighty-eight women were included in the final analysis, of which 38 (43.19%) were diagnosed with DU and 50 (56.81%) with BOO. The median age of the patients with BOO was 56 (42–64) and of those with DU 59.5 (44–69). The main presenting symptom in 27 (30.68%) patients with DU was urgency, while among patients with BOO it was both urgency and nocturia, in 37 (42.05%) cases. In the CLSS questionnaire, the DU group recorded the highest median scores for frequency (2 points; range 0–2), nocturia (2 points; range 1–2), and urgency. In contrast, the BOO group’s highest score was for slow stream, with a mean of 3 points (range 0–3). The detailed symptoms and CLSS questionnaire scores are presented in Table 1.

Regarding chronic diseases, within the BOO group, diabetes mellitus (DM) was present in four (4.55%) patients and hypothyroidism in six (6.82%) patients. However, in the DU group, these comorbidities were present in three (3.41%) and six (6.82%) patients, respectively. Moreover, only two patients (2.27%) in the DU group used cholinolytics and none used alpha-blockers. In the BOO group, six patients (6.82%) were administered cholinolytics and three (3.41%) alpha-blockers. The drugs were continued until the urodynamic study. The continuation of baseline characteristics, including comorbidities and medications used, is presented in Supplementary Table S1.

On UFL, patients with DU had a median Qmax of 15.5 mL/s (10.9–20.1), a median voided volume of 274.5 (207–446), and a median time to Qmax of 15 s (6–26). Patients diagnosed with BOO demonstrated a similar median Qmax of 16.6 mL/s (11.6–20.5), similar median voided volume of 279 mL (IQR 202–406), and a shorter median time to Qmax of 9 s (6–13). The predominant UFL curve shapes observed in DU patients were fluctuating in 18 (20.45%) cases and fluctuating-intermittent in 13 (14.77%), mirroring the prevailing shapes in the BOO group (*n* = 21, 23.86%, *n* = 17, 19.32%, respectively). The median PIP1 in the DU group was 14.25 (7.4–21.9) and in the BOO group, 62.9 (49.7–89.7), while BOOIf measured 3.2 (0–7) in the DU patients and 34.8 (20.4–60) in the BOO women. Detailed urodynamic findings including the patterns of urine flow and detrusor voiding pressure curves are presented in Table 2.

### 3.2. Uni- and Multivariate Logistic Regression Analyses

In the univariate logistic regression analysis, only the time to Qmax on UFL (OR = 1.03, 95% CI 1.00–1.06, *p* = 0.04) and POP-Q < 3 on physical examination prior to UDS (OR = 4.10, 95% CI 1.08–15.62, *p* = 0.04) were statistically significant predictors of DU (*p* < 0.05). Complete univariate analyses for the prediction of DU are demonstrated in Table 3.

For the multivariate analysis, variables that demonstrated statistical significance or were on the threshold of significance in the univariate analyses (*p* < 0.2) were selected. Those parameters included hesitancy, incomplete emptying, straining points in CLSS, time to Qmax on UFL, and POP-Q < 3 in the clinical assessment. The final predictive model comprised three key factors: clinical symptoms, a UFL parameter, and the POP-Q assessment (Table 4).

The model indicated that the occurrences of hesitancy (OR = 2.06, 95% CI 0.71–5.98, *p* = 0.18) and incomplete emptying (OR = 3.52, 95% CI 1.27–9.79, *p* = 0.016), POP-Q < 3 (OR = 0.15, 95% CI 0.03–0.75, *p* = 0.02), and longer time to Qmax on UFL (OR = 1.05, 95% CI 1.02–1.09, *p* = 0.004) were significant predictors of DU diagnosis. The risk model incorporating these predictive factors for DU diagnosis in female patients with non-neurogenic LUTS due to DU or BOO achieved a Harrell’s Concordance Index (C-index) of 0.78. To assess the model’s diagnostic accuracy, a probability cutoff of 0.3 was applied. The resulting clinical diagnostic criteria for urodynamically confirmed DU exhibited a sensitivity of 86.8%, specificity of 46%, positive predictive value (PPV) of 55%, and negative predictive value (NPV) of 82.1%.

## 4. Discussion

The present study promotes the hypothesis that the evaluation of clinical symptoms, UFL analysis, and pelvic organ prolapse assessment using the POP-Q scale can serve as a non-invasive method for differentiating DU from BOO in female patients with LUTS. Our analysis led to the development of a predictive model indicating that hesitancy, incomplete emptying, prolonged time to Qmax on UFL, and POP-Q stage < 3 are significant predictors of DU, rather than BOO, in women with non-neurogenic LUTS. To our knowledge, this study is the first to employ PIP1 < 30, as currently recommended by ICS-SUFU [5], as a diagnostic criterion for DU in females. Additionally, it is the first to integrate a POP-Q assessment into a non-invasive model to distinguish between BOO and DU.

The proposed model incorporates clinically accessible parameters that do not require advanced diagnostic tools, beyond standard UFL, and can be assessed without catheterization. This is particularly relevant for elderly patients or those with comorbidities, who may be unable or unwilling to undergo UDS, or who may have difficulty cooperating during the procedure, potentially leading to biased or inconclusive results. Here, the rather young cohort tested had a median age of 56 years. Yet, a non-invasive approach improves patient accessibility and compliance, while also reducing the time and costs associated with diagnosis in all age groups. Early and accurate differentiation between DU and BOO is critical, as timely intervention can prevent complications such as chronic urinary retention, recurrent infections, and upper urinary tract damage. By offering a practical, efficient, and cost-effective diagnostic tool, this model has the potential to assist clinical decision-making, guide the need for invasive UDS, and optimize healthcare resource utilization.

Due to similarities between the symptoms of DU and BOO, both in men and women, it remains impossible to reliably differentiate between these two disorders based solely on the voiding or storage symptoms [14,15]. A study by D’Alessandro et al. demonstrated that in women attending a urogynecology clinic, among others, voiding/bulging symptoms and anterior and central prolapse were correlated positively with DU, whereas stress urinary incontinence showed an inverse relationship. However, the final diagnostic models exhibited poor accuracy across all evaluated definitions of DU, with the area under the curve (AUC) ranging between 0.64 and 0.72 [16]. Contrary to these findings, our study indicates that clinical symptoms, specifically hesitancy and incomplete emptying, can become valuable predictive factors once integrated into a broader diagnostic model. Although, to our knowledge, no previous studies specifically investigated a symptom-based model differentiating between DU and BOO in females, research in males by Gammie et al. observed incomplete emptying significantly more frequently in DU patients [17], supporting our findings. Nevertheless, it is important to acknowledge that self-reported symptoms are inherently subjective and may introduce variability between patients.

Interestingly, and in alignment with previous observations by Rubilotta et al. our analysis identified a POP-Q stage lower than 3 as a significant predictor of DU. Existing research has demonstrated a high prevalence (40.9%) of DU among patients with pelvic organ prolapse (POP), especially highlighting more severe cystocele in those with DU [18]. Moreover, another study reported that DU was present in approximately half of women undergoing urodynamic evaluation for symptomatic POP, with the likelihood of DU diagnosis increasing substantially at advanced POP stages (stage 4) [18]. Our findings demonstrated that rather mild-to-moderate prolapse (POP-Q < 3) might indicate impaired detrusor function rather than BOO.

UFL has also been extensively studied as a non-invasive diagnostic method to distinguish different types of VD among patients with LUTS. Cheng et al. identified key UFL parameters—Qmax, flow time, and voiding efficiency—as independent risk factors for female BOO in women undergoing UDS; however, they did not explicitly evaluate DU patients [19]. Another study specifically examining DU predictors highlighted prolonged time to Qmax (greater than 13.5 s), altered mean flow ratios, and extended overall flow times as indicators of detrusor impairment, which align closely with our findings, though in a different patient population [20]. Furthermore, one study showed that among free-flow parameters, lower Qmax and higher bladder voiding efficiency were more predictive of BOO compared to DU in a multivariate regression analysis [21]. Our results complement these observations, reinforcing the concept that certain UFL-derived metrics, specifically the prolonged time to Qmax, can reliably support the differentiation between DU and BOO, particularly when interpreted within a structured diagnostic framework.

The diagnostic utility of flow-curve morphology remains more controversial. A recent 2020 study found that bell-shaped flow patterns effectively excluded BOO, whereas prolonged curves primarily indicated obstruction rather than DU. Fluctuating and intermittent curves were associated equally with both conditions, thereby limiting their diagnostic utility for clearly distinguishing between DU and BOO [22]. Another analysis indicated that prolonged or tailed curves were independent predictors for BOO when differentiating from DU or undetermined urodynamic findings (unclassified PFS) [23]. In contrast, in our current study, flow-curve shape did not emerge as a significant differentiating predictor, possibly due to the relatively limited sample size. Surprisingly, and in contrast with previous studies [2,19,24], patient age was not found to be a significant predictor of DU in our cohort. While age-related decline in detrusor function is well documented, the relatively young median age of our population (median 56 years; IQR 42–64) may explain this discrepancy.

Our study has several limitations that must be acknowledged. First, as a retrospective study, it is inherently subject to selection bias. Second, the relatively small sample size (*n* = 88) may limit statistical power and increase the risk of model overfitting, particularly in the context of a multivariate model with four predictors. While the variables were selected based on both statistical thresholds and clinical plausibility, the robustness of the model remains uncertain due to the limited sample size. Third, the absence of external validation further restricts the generalizability of our findings. The model has not yet been tested in an independent cohort, which is essential to establish its clinical utility. We therefore consider this study to be a pilot investigation aimed at generating hypotheses and informing the design of future validation studies. Another limitation is the lack of data on menopausal status and hormonal therapy, both of which are known to influence detrusor function and LUTS in women. Although medication use (e.g., anticholinergics, alpha-blockers) was recorded, it was not adjusted for in the regression analysis due to heterogeneity and limited subgroup sizes. These unaccounted confounders may have affected the observed associations. Finally, the study population consisted entirely of patients from a single geographic region and ethnic background, which may limit the applicability of our findings to more diverse populations. Future research should focus on prospective validation in larger, multi-center cohorts with broader demographic representation and the incorporation of additional clinical variables to enhance diagnostic accuracy.

## 5. Conclusions

In summary, we developed a non-invasive prediction model that integrates easily available clinical parameters—hesitancy, incomplete emptying, POP-Q assessment, and time to Qmax on UFL—to assist in differentiating DU from BOO in female patients with LUTS. While many prior attempts at symptom-based or single-parameter differentiation proved insufficient, our combined approach shows promise in terms of diagnostic value and clinical applicability and may serve as a supportive diagnostic aid in selected patients. Future studies in larger and more diverse populations are warranted to prospectively validate and refine the model.

## Figures and Tables

**Table 1:** Baseline characteristics of included patients with detrusor underactivity (DU) and bladder outlet obstruction (BOO).

			BOO (*n* = 50)		DU (*n* = 38)		
			No. of Patients/Median	% of Patients/IQR	No. of Patients/Median	% of Patients/IQR	*p*-Value
Age		Years	56	42–64	59.5	44–69	0.22
Symptoms	Urgency		37	42.05	27	30.68	0.76
	Frequency		33	37.50	24	27.27	0.78
	Nocturia		37	42.05	24	27.27	0.27
	Weak stream		21	23.86	19	21.59	0.46
	Hesitancy		11	12.50	14	15.91	0.13
	Intermittency		11	12.50	7	7.95	0.68
	Straining		5	5.68	7	7.95	0.25
	Incomplete emptying		17	19.32	20	22.73	0.08
	Dribble		0	0	1	1.14	0.25
	UUI		23	26.14	21	23.86	0.39
	SUI		25	28.41	16	18.18	0.46
	Pads (daytime)		0	0–3	0	0–3	0.36
	Pads (nighttime)		0	0–0	0	0–0	0.62
CLSS questionnaire	Frequency	points	2	0–2	2	0–2	0.74
	Nocturia	points	2	1–2	2	1–2	0.62
	Urgency	points	2	0–2	2	1–3	0.32
	Slow stream	points	3	0–3	0	0–3	0.35
	Straining	points	0	0–0	0	0–0	0.16
POP assessment	POP-Q < 3		37	42.05	35	39.77	0.03
Delivery method	Vaginal birth		2	1–2	2	0–2	0.10
	Cesarean section		0	0–0	0	0–0	0.44

CLSS—Core Lower Urinary Tract Symptoms score, POP—pelvic organ prolapse, UUI—urge urinary incontinence, SUI—stress urinary incontinence.

**Table 2:** Comparison of urodynamic findings in patients with DU and BOO.

			BOO (*n* = 50)		DU (*n* = 38)		
UFL Parameters	Qmax	mL/s	No. of Patients/Median	% of Patients/IQR	No. of Patients/Median	% of Patients/IQR	*p*-Value
	Voided volume	mL	16.6	11.6–20.5	15.5	10.9–20.1	0.65
	Qav	mL/s	279	202–406	274.5	207–446	0.97
	Qmax–Qav difference	mL/s	6.8	5.3–9.8	6.3	5.5–9.3	0.65
	Voiding time	s	8	5.7–11.4	7.5	5.9–10.4	0.7
	Time to Qmax	s	47.5	29–74	44	35–64	0.99
	PVR	mL	9	6–13	15	6–26	0.06
	PVR ratio		17.5	10–100	45	10–200	0.25
UFL curve shapes	Bell-shape		0.07	0.03–0.26	0.09	0.04–0.34	0.17
	Fluctuating		8	9.09	6	6.82	0.98
	Intermittent		21	23.86	18	20.45	0.62
	Fluctuating-Intermittent		0	0	0	0	
	Plateau		17	19.32	13	14.77	0.98
Pressure-flow study parameters	Qmax	mL/s	13	14.77	8	9.09	0.59
	Pdetmax	cm H_2_O	9.7	7.4–12.5	3.45	0–7.9	<0.0001
	Pdet@Qmax	cm H_2_O	64	51–113	12.5	7–21	<0.0001
	PIP1		54.5	40–80	9	7–18	<0.0001
	BOOIf		62.9	49.7–89.1	14.25	7.4–21.9	<0.0001
			34.8	20.4–60	3.2	0–7	<0.0001

UFL—uroflowmetry, PVR—post-void residual urine, Qmax—maximum flow, Qav—mean flow, Pdetmax—maximum detrusor pressure, Pdet@Qmax—detrusor pressure at maximum flow, PIP1—projected isovolumetric pressure 1, BOOIf—Female Bladder Outlet Obstruction Index.

**Table 3:** Univariate logistic regression analyses of factors predictive for DU in female patients with non-neurogenic LUTS with DU or BOO diagnosed on a PFS.

	Detrusor Underactivity					
Variable				OR	0.99–1.04	0.32
Age	Urgency		yes vs. no	1.01	0.34–2.21	0.76
Symptoms	Frequency	Urgency	yes vs. no	0.86	0.37–2.13	0.78
	Nocturia	Frequency	yes vs. no	0.88	0.24–1.50	0.28
	Weak stream	Nocturia	yes vs. no	0.60	0.59–3.23	0.46
	Hesitancy	Weak stream	yes vs. no	1.38	0.81–5.29	0.13
	Intermittency	Hesitancy	yes vs. no	2.07	0.28–2.31	0.68
	Straining	Intermittency	yes vs. no	0.80	0.59–6.99	0.26
	Incomplete emptying	Straining	yes vs. no	2.03	0.91–5.12	0.08
	Dribble	Incomplete emptying	yes vs. no	2.16	0.001–999.99	0.99
	UUI	Dribble	yes vs. no	>999.99	0.62–3.38	0.39
	SUI	UUI	yes vs. no	1.45	0.31–1.70	0.46
	Pads (daytime)	SUI	points	0.73	0.66–1.43	0.87
CLSS questionnaire	Pads (nighttime)	Frequency	points	0.97	0.74–1.67	0.62
	Frequency	Nocturia	points	1.11	0.78–1.76	0.44
	Nocturia	Urgency	points	1.17	0.71–1.73	0.64
	Urgency	UUI	points	1.11	0.78–1.60	0.56
	Slow stream	SUI	points	1.11	0.65–1.16	0.34
	Straining	Slow stream	points	0.87	0.88–1.99	0.18
	POP-Q < 3	Straining	points	1.32	0.81–1.45	0.60
		Incomplete emptying	continuous	1.08	0.95–1.05	0.90
UFL		Qmax	continuous	0.99	0.99–1.00	0.77
		Voided volume	continuous	1.0	0.87–1.08	0.58
		Qav	continuous	0.97	0.94–1.09	0.84
		Qmax–Qav difference	continuous	1.01	0.99–1.01	0.63
		Voiding time	continuous	0.99	1.00–1.06	**0.04**
		Time to Qmax	continuous	1.03	0.99–1.00	0.49
		PVR	continuous	1.00	0.37–24.82	0.30
		PVR ratio	yes vs. no	3.02	0.31–3.12	0.99
UFL curve shapes		Bell-shape	yes vs. no	0.98	0.53–2.91	0.62
		Fluctuating	yes vs. no	1.24	0.42–2.46	0.98
		Fluctuating-intermittent	yes vs. no	1.01	0.28–2.07	0.59
	Vaginal birth	Plateau	yes vs. no	0.76	1.08–15.62	**0.04**
POP-Q scale	Cesarean section	POP-Q < 3	yes vs. no	4.10	0.21–4.69	0.99
		DM	yes vs. no	0.99	0.14–5.49	0.88
Chronic diseases		Recurring UTIs	yes vs. no	0.87	0.24–31.19	0.42
		Hashimoto	yes vs. no	2.72	0.41–4.66	0.61
		Hypothyroidism	yes vs. no	1.38	95% CI	*p*-value

CLSS—Core Lower Urinary Tract Symptoms score, UFL—uroflowmetry, PVR—post-void residual urine, Qmax—maximum flow, Qav—mean flow, DM—diabetes mellitus, POP-Q—Pelvic Organ Prolapse-Quantification System, UUI—urge urinary incontinence, SUI—stress urinary incontinence.

**Table 4:** A multivariate logistic regression analysis for predicting DU in female patients with non-neurogenic LUTS.

Detrusor Underactivity					
	Variable		OR	95% CI	*p*-Value
Symptoms	Hesitancy	0	ref	-	
		1	2.06	0.71–5.98	**0.18**
	Incomplete emptying	0	ref	-	
		1	3.52	1.27–9.79	**0.02**
POP-Q scale	POPQ < 3	0	ref	-	
		1	0.15	0.03–0.75	**0.02**
UFL parameters	Time to Qmax		1.05	1.02–1.09	**0.004**

UFL—uroflowmetry, POP-Q—Pelvic Organ Prolapse-Quantification System, Qmax—maximum flow.

## Data Availability

The data presented in this study are available upon request from the corresponding author.

## Supplementary Materials

The following supporting information can be downloaded at: https://www.mdpi.com/article/10.3390/jcm14113674/s1. Supplementary Table S1. Baseline characteristics of included patients with detrusor underactivity (DU) and bladder outlet obstruction (BOO), continuation. Supplementary Table S2. Univariable analysis—continuation.

## Abbreviations

The following abbreviations are used in this manuscript:

DU	Detrusor Underactivity
VD	Voiding Dysfunction
LUTS	Lower Urinary Tract Symptoms
BOO	Bladder Outlet Obstruction
UDS	Urodynamic Studies
ICS	International Continence Society
SUFU	Society of Urodynamics, Female Pelvic Medicine & Urogenital Reconstruction
BCI	Bladder Contractility Index
ICS-DCI	ICS-Detrusor Contractility Index
DVC	Detrusor Voiding Contraction
D[B]CI	Detrusor/Bladder Contractility Index
PIP1	Projected Isovolumetric Pressure 1
BOOIf	Female-Specific Bladder Outlet Obstruction Index
BOOI	Bladder Outlet Obstruction Index
ICS-PFS	Provisional ICS Pressure-Flow Study (Nomogram)
CLSS	Core Lower Urinary Tract Symptoms (questionnaire)
POP-Q	Pelvic Organ Prolapse Quantification
PFS	Pressure-Flow Studies
UFL	Urine Flow Test (Uroflowmetry)
PVR	Post-Void Residual
PVR-R	Post-Void Residual Ratio
IQR	Interquartile Range
OR	Odds Ratio
CI	Confidence Interval
PPV	Positive Predictive Value
NPV	Negative Predictive Value
