# Supplementary material for: Cracking the LUTS Code: A Pre-Urodynamic Tool for DU vs. BOO Diagnosis in Female Patients with Non-Neurogenic LUTS
Source: J Clin Med. 2025 May 23;14(11):3674. doi: 10.3390/jcm14113674 (PMC12156388; doi:10.3390/jcm14113674)
Supplement: Supplementary file 1 [file jcm-14-03674-s001.zip › jcm-3571360-supplementary.pdf]

Supplementary Table S1. Baseline characteristics of included patients with detrusor underactivity (DU) and bladder outlet obstruction (BOO), continuation.

			BOO (n=50)		DU (n=38)		
			No. of patients/ median	% of patients/ IQR	No. of patients/ median	% of patients/ IQR	P-value
CLSS questionnaire cont.	Incomplete emptying	points	0	0-3	0.5	0-3	0.61
	Stress incontinence	points	0.5	0-2	0	0-3	0.64
	Urge incontinence	points	0	0-2	1	0-2	0.65
	CLSS sum	points	9.5	7-13	11	7-14	0.56
Chronic diseases	DM		4	4.55	3	3.41	0.99
	Recurring UTIs		3	3.41	2	2.27	0.88
	Hypothyroidism		6	6.82	6	6.82	0.61
	Hashimoto		1	1.14	2	2.27	0.40
	Aortic Sclerosis		0	0	1	1.14	0.25
Drugs	Cholinolytics		6	6.82	2	2.27	0.28
	Alpha-blockers		3	3.41	0	0	0.12
	Insulin		1	1.14	0	0	0.38
	Oral drugs for DM		4	4.55	2	2.27	0.61
	Statins		5	5.68	2	2.27	0.42
Past surgeries	Hysterectomy		4	4.55	6	6.82	0.25
	TOT/TVT		9	10.23	4	4.55	0.33

* Cont. - continuation, CLSS - Core Lower Urinary Tract Symptoms score, DM - diabetes mellitus, UTI - urinary tract infection, TOT - Transobturator Tape, TVT – Transvaginal Tape

Supplementary Table S2. Univariable analysis – continuation.

Variable			OR	95% CI	P-value
CLSS questionnaire	Painful bladder	points	0.71	0.42-1.81	0.87
	Painful urethra	points	0.01	0.001-999.99	0.99
	Overall CLSS score	points	1.03	0.93-1.14	0.57
	Statins	yes vs no	0.50	0.09-2.73	0.42
	Neuroleptics	yes vs no	1.32	0.08-21.88	0.84
	Distygmine	yes vs no	2.72	0.24-31.19	0.42
	Mirabegron	yes vs no	0.42	0.04-4.24	0.46
	Cholinolytics	yes vs no	0.41	0.08-2.14	0.29
Drugs	Insulin	yes vs no	<0.001	<0.001->999.99	0.99
	Oral drugs for DM	yes vs no	0.64	0.11-3.69	0.62
	Levothyroxine	yes vs no	1.66	0.51-5.41	0.40

* CLSS - Core Lower Urinary Tract Symptoms score, DM – diabetes mellitus,
